# To vaccinate or not to vaccinate? Women’s perception of vaccination in pregnancy: a qualitative study

**DOI:** 10.3399/bjgpopen18X101457

**Published:** 2018-04-04

**Authors:** Aisling O'Shea, Brian Cleary, Edel McEntee, Tina Barrett, Austin O'Carroll, Richard Drew, Fiona O'Reilly

**Affiliations:** 1 GP Graduate, GP Training Scheme, North Dublin City GP Training Programme, Dublin, Ireland; 2 Lead Pharmacist, Pharmacy Department, The Rotunda Hospital, Dublin, Ireland; 3 GP Graduate, GP Training Scheme, North Dublin City GP Training Programme, Dublin, Ireland; 4 Pharmacist, Pharmacy, The Rotunda Hospital, Dublin, Ireland; 5 Head of the NDCGP Training Scheme, GP Training Scheme, North Dublin City GP Training Programme, Dublin, Ireland; 6 Microbiologist, Microbiology Department, The Rotunda Hospital, Dublin, Ireland; 7 Programme Director, GP Trainings Scheme, North Dublin City GP Training Programme, Dublin, Ireland

**Keywords:** vaccination, pregnancy, qualitative, influenza, pertussis

## Abstract

**Background:**

Vaccination against influenza and pertussis in pregnancy can reduce the significant morbidity and mortality associated with these infections. Despite this, there is poor uptake of both vaccines in pregnancy.

**Aim:**

To explore women’s perception of vaccination in pregnancy and thereby determine the reasons behind such low vaccination rates.

**Design & setting:**

This is a qualitative study undertaken at a large maternity hospital.

**Method:**

Seventeen post-partum women completed a semi-structured interview discussing vaccination. They were recruited from a quantitative study looking at vaccination rates in pregnancy. The interview transcripts were discussed among three researchers and underwent thematic analysis.

**Results:**

Three themes emerged. The first theme explored the influencing factors that shaped the women’s decision to vaccinate in pregnancy. The recommendation of a healthcare provider was the most important influencing factor for this study's cohort of women. The second theme highlighted the deficiency in knowledge women had regarding vaccine safety. The last theme related to the pertussis vaccine, and the reluctance of healthcare providers to discuss and offer this vaccine in pregnancy.

**Conclusion:**

The qualitative approach gives voice to the thoughts and concerns of women as they make the complex decision to vaccinate in pregnancy. Clinicians must be cognizant of the important role they play in advising women to vaccinate in pregnancy. They must advise women that the vaccine is safe and address any of their concerns. Lastly, a message on vaccine safety should be included in future public health campaigns to promote vaccination in pregnancy.

## How this fits

There are numerous quantitative studies linking demographic factors with low vaccination rates in pregnancy. This study takes a qualitative approach from an under-researched geographical location and gives an insight into pregnant women’s concerns and influencing factors as they make the complex choice to vaccinate. This information could be used by healthcare providers involved in maternity care to improve vaccination rates in pregnancy.

## Introduction

Influenza and pertussis cause significant morbidity and mortality during pregnancy and early infancy.^1^ Pregnant women with influenza are more likely to be hospitalised and admitted to an intensive care unit compared with the general population.^2^ Between 2009–2012 in the UK, there were an estimated 11 deaths per 100 000 pregnant women, with influenza responsible for 1 of these deaths.^3^ Maternal influenza infection is also associated with complications for the fetus including miscarriage, preterm delivery, low birth weight, congenital anomalies, and fetal death.^2,4,5^ Similarly, pertussis infection in young infants is often severe and potentially fatal.^6,7^ The Public Health Authority UK reported 14 deaths from pertussis in children aged <3 months in 2012. Vaccination against influenza and pertussis is recommended in pregnancy to reduce this morbidity and mortality.^8–10^ Both vaccines have been extensively studied and are considered safe for both mother and fetus in pregnancy.^5,11–13^ Despite these well-documented benefits and safety data, there is poor uptake of both vaccines among pregnant women.^1,14–17^

Public Health England estimated that only 40.3% of pregnant women had the seasonal flu vaccine in 2012/2013,^14^ and they estimate pertussis vaccine uptake at 54% in 2014.^16^

There have been numerous quantitative studies investigating the factors affecting vaccination rates.^18–20^ Conversely, there have been relatively few qualitative studies focusing on mothers’ interpretation of influences on their choices about vaccination in pregnancy.^1^ Existing qualitative literature comes from locations with diverse cultures, including Hong Kong,^21^ North America,^22,23^ Brazil,^24^ Australia,^25^ and Morocco.^26^ The present authors found only one qualitative study exploring this topic from the UK and Ireland, and this was conducted during the 2009 pandemic.^27^ Recent literature agrees on the importance of the healthcare provider’s advice and raises concerns over the lack of safety information.^21,28^ However, the importance of family and cultural influence in maternal decision-making has also been found in different cultural settings.^24,26,29^ This study aims to build on previous qualitative work in a new study location (Ireland), using qualitative interviews to understand influences to maternal decision-making.

## Method

### Participants

The study was nested in a wider mixed-methods study that recruited 198 women from a tertiary referral maternity hospital in Dublin, Ireland, to determine the vaccination rates of influenza and pertussis in pregnancy. The study also engaged 1189 healthcare providers in a postal survey to determine their knowledge, attitudes, and practice around influenza vaccination in pregnancy. The study took place between January and June 2016.

Hospital admissions were eligible for inclusion if the patient had a live birth at >24 weeks, was aged >18 years, and could understand and answer the quantitative survey in English. After completing a questionnaire, participants were purposively selected to achieve maximum variation in terms of vaccination status, public or private patient status, and ethnicity. Women meeting the criteria were asked to take part in a telephone interview within 1 month of delivery. The women were consented for the qualitative arm, and their name and telephone number were passed on to the qualitative interviewer.

Initially, 42 women consented to the interview. An attempt was made to contact all these women by telephone, some on multiple occasions. Though data saturation had been achieved, there were only two unvaccinated women among those interviewed. A further 18 women were consented, but only unvaccinated women were selected for interview.

### Interviews

The telephone interview was chosen instead of face-to-face due to the limited time the women had available while on the post-delivery ward. The phone interview within 1 month of delivery would allow a more relaxed conversation with the recent mothers at times most suited to their schedule. This was achieved by arranging suitable call-back times. A total of 17 in-depth interviews were carried out by a single interviewer. A semi-structured questionnaire was used to guide the interview.

The design of the interview was structured to allow multiple participants answer the same questions, thus facilitating saturation. The interview guide was created by firstly identifying the general domains the study wanted to explore; that is, the women’s knowledge and awareness of vaccination guidelines, their understanding of influences, and their views of benefits and risks of vaccination during pregnancy. Open questions were then determined within these domains to allow for interpretations of their experiences that influenced their views. Following Silverman,^30^ the interviewer was able to add extra questions about unexpected but relevant responses that emerged (full questionnaire available from the authors on request).

### Semi-structured questionnaire

All the interviews were recorded and transcribed by two members of the research team. A separate researcher checked the transcriptions for accuracy and anonymised the transcripts before analysis.

### Analysis

The transcripts were analysed collaboratively using a thematic approach, using the qualitative research interview to understand the world from the subjects’ point of view prior to scientific explanations.^31^ Two of the research team read a subset of the transcripts and generated initial codes. A coding template was defined using these, and codes developed from the interview guide in MAXQDA software (version 12). All transcripts were imported and coded; free codes were assigned if pre-defined codes did not suffice. Codes were examined for themes which were reviewed, defined, and refined. A second party reviewed coding of transcripts to ensure data saturation had been reached.^32^ Saturation was determined as 'no new data or themes emerging'.^33^ A third member of the research team, having read all the transcripts, reviewed the codes and themes. Subanalysis was conducted to see if there were differences according to different ethnicity, public or private status, and vaccination status.

## Results

Seventeen women participated in a semi-structured interview by a single interviewer. The women were aged 23–44 years. The average age was 33 years. The majority of women were Irish (*n* = 12), although there was one Polish, one Nigerian, one Spanish, one Maltese, and one Pakistani participant, reflecting the varying ethnicities admitted to the maternity hospital. Five of the women were seen privately for their maternity care, four semi-privately, and eight under the general medical service scheme (which is the equivalent to the NHS). The interviews took between 7 mins 35 seconds and 20 mins 14 seconds; the mean length of an interview was 12 mins 26 seconds. All interviews were successful in covering the topics with the level of detail required to inform the research objective. Thirteen of the 17 women received the influenza vaccine in pregnancy, while only nine received the pertussis vaccine. Three themes emerged from the transcripts. Differences were found relating to vaccination status and these are noted below. No strong differences were found in other subgroups; however, numbers within these subgroups were low.

### Theme one: healthcare providers influence pregnant women’s choice to vaccinate

Recommendation to vaccinate from a healthcare provider emerged as a very important factor influencing the women’s decisions to vaccinate. Twelve of the 13 women vaccinated were positively influenced by their GP, midwife, or hospital consultant to have the influenza vaccine. The women felt it was important to follow the advice of their healthcare provider and some took a passive role in seeking any further information regarding the influenza vaccine once it was recommended to them by their healthcare provider:

*‘I wouldn’t spend an awful lot of time reading about it, if it was a recommended vaccine I’d just get it.’* (Interview 2)

*‘I think it’s important to listen to the advice of your GP and your obstetrician because they’re the ones that know best, you know, when it comes to anything in pregnancy. They’ve seen it all, I guess.’* (Interview 15)

The one woman who did not cite a healthcare provider as a positive influence had a medical background and consulted the Public Health Authority website herself for current recommendations.

In contrast, two of the unvaccinated group said that they were not offered the vaccine by their healthcare provider. They did not discuss the merits or safety of the vaccine with any healthcare provider. Others among the vaccinated group commented that the vaccine was not stressed enough and that healthcare providers did not engage them in meaningful discussions about vaccination:

*‘But, like, it’s not really stressed as much as it probably should be I think. It is kinda a big deal, I know it’s your decision and all but it’s not really stressed as much as it probably should be, we shouldn’t have the choice to say ya or no.’* (Interview 15)

Another unvaccinated woman (Interview 4) explained that the vaccine was not ‘*pushed too much’,* that it was left to her to consider it herself, and that she was *‘left to (her) own devices.’* One woman believed that the vaccines were not part of routine antenatal, as other items such as anti-D prophylaxis were; she did not see vaccination as part of the pregnancy plan:

*‘So I think giving them a plan at the start that it would be additional, you know the way, when you go and you get your booking visit and they check your blood group and they say, you know, "You’re rhesus positive and you should get anti-D" and all. You know, I got that plan from very early on. They booked me and everything, so in the same way, you know, the patient should be told: these two injections, like vaccinations, you should get and then you know.’* (Interview 14)

The women discussed access to the vaccine. They commended its availability at their community GP visits but they repeatedly commented that it was not discussed enough in the hospital environment either by the midwives or by their consultant obstetrician:

*‘I received the information, I learned about the vaccine at the GP not in the hospital so maybe the doctors in the hospital could tell people about the vaccine.’ (*Interview 6)

Four women described stories that negatively influenced their decision to vaccinate or caused them to proceed with more caution. Some referred to the bad press surrounding certain vaccines, such as the link between the H1N1 vaccine and narcolepsy, and the (completely discredited) link between autism and the MMR vaccine:

*‘It’s a story but it’s a real life story, it’s the truth, so I mean that girl is crucified for the rest of her life now, and it’s not just her and I know because her mum is in a group of mums and dads who are trying to bring a case to the government, to answer for the result the effect this vaccine has had on all of the children. There’s a whole group of them, it’s not just one person; there’s a whole movement that has been affected by narcolepsy in particular with relation to the swine vaccine.’ (*Interview 10)

A number of women discussed relatives or people they knew who received the flu vaccine who subsequently contracted the flu or got the flu despite the vaccine. The story that the vaccine can give you the flu negatively influenced two of the unvaccinated group:

*‘But, eh, my father had it for the last 4 or 5 years and he’s contracted the cold and the flu even though he’s had it, so I don’t see the point in taking it.’ (*Interview 13)

*‘My sister in law is school teacher, both of them are, my brother who is married to her, both of them are primary school teachers and both of them got the flu vaccine and both of them got the flu straight away. A version of the flu, but it was very bad and it was frustrating for both of them and then they say "That’s it, they are immune now", well immune 'til next year when they get it again and they get the flu again. I just think that’s ridiculous. I never heard anything like it in all my life.’* (Interview 10)

Family and friends were not seen as a major influence to decision-making about vaccination but were seen to be largely positive.

### Theme two: the lack of understanding regarding vaccine safety

This theme was unexpected; it had been postulated that the hesitancy towards vaccination would stem from the perceived risks or safety concerns surrounding vaccination in pregnancy. Interestingly, after speaking to the women, some admitted somewhat hesitantly that if the vaccine was recommended to them by their healthcare provider they did not investigate it further or consider the safety of the vaccine. However, over half did engage in some form of deliberation. When asked if they knew any risks associated with the influenza vaccine, over half of the women were unsure whether there were any risks associated with the influenza vaccine:

‘*Well, mmm, not too much now* [knowledge about risk and safety]*, because there is a lot of controversy* [pause] *I wouldn’t know what are the risks of the normal vaccination in children are or the flu vaccination but I trust in them because I think they are being used before a long time, they are working pretty well, so I don’t read about what are the risks because I am not afraid of that.’* (Interview 6)

In the unvaccinated group, the perceived risks associated with the vaccine discouraged two of them from receiving it, as illustrated below. However, the remaining women were uncertain of the risks or safety:

*‘I think the risks of vaccination in pregnancy are huge. I think if you interfere with pregnancy at all, you’re asking for trouble.’ (*Interview 10)

Women believed that there were risks associated with the influenza vaccine but that they had a poor understanding of what these risks were. There was a lack of awareness of the safety of the influenza vaccine, with only three women being confident that there is no risk to the baby if the mother receives the influenza vaccination in pregnancy.

### Theme three: the lack of awareness and promotion of the pertussis vaccination

It was expected that when asked about the pertussis vaccine in pregnancy, women would raise concerns regarding its safety, particularly since it is a relatively new vaccine. In fact, it transpired that the pertussis vaccine was less likely to be discussed with pregnant women. Seven women interviewed had never heard of the pertussis vaccine. For many who did receive the vaccine, they explained their healthcare provider was much less involved in giving information on this vaccine compared to the influenza vaccine, and these women sought other sources of information to inform their choice. Several had to engage their healthcare provider in discussion about the vaccine:

'*I was reading on a website and I had to go and ask for it myself, that was it. So nobody mentioned anything, not even the midwives or anything, I was completely unaware of it and then I happened to read and I went to get it last minute, again at my GP, that one.'* (Interview 11)

The women allude to some ambiguity among healthcare providers over who is responsible for discussing and offering the pertussis vaccine:

*'It was me who brought it to the GP and actually when I said it to the GP, the GP said "I thought this was something the hospitals should be doing." I am not too sure if maybe there is miscommunication between my own GP and the hospital over that. But he did give it.’ (*Interview 8)

During the interview, those women who were not aware of the vaccine were asked if they would consider it now. A number suggested they would:

**Interviewer:** *‘So you think if you were offered it, it would have been something you’d have considered?'*

**Participant:** *'Ye ye, if it was suggested by the doctor yeah.’* (Interview 16)

## Discussion

### Summary

Similar to the results found in other studies,^1,14–17^ the vaccination rates in the quantitative arm of this study were found to be low for influenza and lower still for pertussis (O'Shea *et al*, unpublished data, 2018). The qualitative arm of the study offers explanations for these suboptimal vaccination rates through describing, postpartum, women’s own views and influences on their decision-making. In brief, the results show that the recommendation of a healthcare provider is the most important influencing factor on a woman’s choice to vaccinate. Knowledge about vaccine safety was lacking, as was awareness of recommendations about the pertussis vaccine.

### Strengths and limitations

A single interviewer was used to standardise interviewer technique, although this comes with its own biases. A collaborative data analysis approach sought to reduce this potential bias. The interviewer was a medical doctor, which may have influenced the data captured. An open, non-judgmental approach was used to guard against this.

This was a single-centered study and did not include women from some of Ireland’s ethnic minorities, including women from the Traveller community and the Roma community, who are known to have particularly low vaccination rates. Furthermore, there was a difficulty recruiting unvaccinated women for the telephone interview and, as a result, they are underrepresented in these results.

The absence of visual cues in telephone interviews is thought to result in loss of contextual and nonverbal data, and to compromise rapport, probing, and interpretation of responses.^34^ Nevertheless, the use of this method allowed interviews to occur at a time and place where the busy new mothers could feel relaxed and, arguably, could disclose sensitive information.

### Comparison with existing literature

Consistent with other studies, these findings highlight the importance of the recommendation of a healthcare provider in positively influencing a pregnant woman’s decision to vaccinate.^21–23,25,28,35^ Similar to the results from Kennedy,^36^ this cohort of women referred to lingering doubts and uncertainties attributed to vaccines in the wake of media reports on risks of vaccination.

There was a discrepancy between the knowledge women had regarding the benefits of vaccinating in pregnancy compared with the lack of knowledge they had regarding vaccine safety. As outlined in other studies, the perceived risks of the influenza vaccine were deterrents to vaccine acceptance for some.^37,38^ However, as found in previous research,^1,28^ many women did not consider the risks of the influenza vaccine if it was recommended to them by their healthcare provider. Both positions of vaccinating and not vaccinating despite uncertainty were found in this study. Similarly, not all vaccinated and unvaccinated women had engaged in deliberation. This suggests that these findings are not fully explained by health behaviour theories^39–41^ that presume deliberation on pros and cons of vaccination using all sources of information available.

Those who did not vaccinate were difficult to recruit for this study. An action research methodology at early stages of pregnancy may elucidate and, indeed, dispel reluctance while contributing to the literature on how this can be more effectively done.

Family and cultural influence in maternal decision-making, which have been found in different cultural settings,^24,26,29^ were not reflected in this study.

Considering the importance women place on healthcare provider recommendation, it will be important for these providers to address and dispel any unfounded safety concerns surrounding the influenza and pertussis vaccine in pregnancy. Given the lack of safety information, the present authors recommend that this is a focus for media campaigns.

### Implications for practice

In Ireland, the influenza vaccine is not available at hospital antenatal appointments. This study suggests that vaccine uptake would increase with a combined effort from both primary care and maternity units, not only promoting the vaccines but making them available at antenatal appointments, whether in the hospital or at the GP clinic. This research shows that women respect healthcare providers and expect them to guide their decisions. Uptake would undoubtedly be increased by having vaccines available at hospital antenatal clinics in addition to primary care, with staff promoting both the influenza and pertussis vaccines and reassuring women about safety.
